# Lasting Changes to Circulating Leukocytes in People with Mild SARS-CoV-2 Infections

**DOI:** 10.3390/v13112239

**Published:** 2021-11-08

**Authors:** Allison E. Kennedy, Laura Cook, Jessica A. Breznik, Braeden Cowbrough, Jessica G. Wallace, Angela Huynh, James W. Smith, Kiho Son, Hannah Stacey, Jann Ang, Allison McGeer, Brenda L. Coleman, Maggie Larché, Mark Larché, Nathan Hambly, Parameswaran Nair, Kjetil Ask, Matthew S. Miller, Jonathan Bramson, Megan K. Levings, Ishac Nazy, Sarah Svenningsen, Manali Mukherjee, Dawn M. E. Bowdish

**Affiliations:** 1 McMaster Immunology Research Centre, McMaster University, Hamilton, ON L8S 4K1, Canada; allkennedy@nosm.ca (A.E.K.); breznikj@mcmaster.ca (J.A.B.); cowbroub@mcmaster.ca (B.C.); wallacjg@mcmaster.ca (J.G.W.); staceyhd@mcmaster.ca (H.S.); ang.jann.catherine@gmail.com (J.A.); mmiller@mcmaster.ca (M.S.M.); bramson@mcmaster.ca (J.B.); 2 Michael G. DeGroote Institute for Infectious Disease Research, McMaster University, Hamilton, ON L8S 4L8, Canada; 3 Department of Medicine, Michael G. DeGroote School of Medicine, McMaster University, Hamilton, ON L8S 4K1, Canada; huynha8@mcmaster.ca (A.H.); smithjw@mcmaster.ca (J.W.S.); sonk@mcmaster.ca (K.S.); mlarche@mcmaster.ca (M.L.); larche@mcmaster.ca (M.L.); hamblyn@mcmaster.ca (N.H.); parames@mcmaster.ca (P.N.); askkj@mcmaster.ca (K.A.); nazyi@mcmaster.ca (I.N.); svennins@mcmaster.ca (S.S.); mukherj@mcmaster.ca (M.M.); 4 BC Children’s Hospital Research Institute, University of British Columbia, Vancouver, BC V5Z 4H4, Canada; lcook@bcchr.ca (L.C.); megan.levings@ubc.ca (M.K.L.); 5 Department of Microbiology and Immunology, The Peter Doherty Institute for Infection and Immunity, University of Melbourne, Melbourne, VIC 3000, Australia; 6 Firestone Institute of Respiratory Health, St. Joseph’s Healthcare, Hamilton, ON L8N 4A6, Canada; 7 Department of Biochemistry & Biomedical Sciences, McMaster University, Hamilton, ON L8S 4K1, Canada; 8 Department of Microbiology, Sinai Health, Toronto, ON M5G 1X5, Canada; allison.mcgeer@sinaihealth.ca (A.M.); Brenda.Coleman@sinaihealth.ca (B.L.C.); 9 Department of Laboratory Medicine and Pathobiology, Faculty of Medicine, University of Toronto, Toronto, ON M5S 1A1, Canada; 10 School of Biomedical Engineering, University of British Columbia, Vancouver, BC V6T 1Z3, Canada; 11 Department of Surgery, University of British Columbia, Vancouver, BC V5Z 1M9, Canada; 12 McMaster Centre for Transfusion Research, Faculty of Health Sciences, Hamilton, ON L8S 4L8, Canada

**Keywords:** COVID-19, SARS-CoV-2, immunophenotype, inflammation, immune activation

## Abstract

Survivors of severe SARS-CoV-2 infections frequently suffer from a range of post-infection sequelae. Whether survivors of mild or asymptomatic infections can expect any long-term health consequences is not yet known. Herein we investigated lasting changes to soluble inflammatory factors and cellular immune phenotype and function in individuals who had recovered from mild SARS-CoV-2 infections (*n* = 22), compared to those that had recovered from other mild respiratory infections (*n* = 11). Individuals who had experienced mild SARS-CoV-2 infections had elevated levels of C-reactive protein 1–3 months after symptom onset, and changes in phenotype and function of circulating T-cells that were not apparent in individuals 6–9 months post-symptom onset. Markers of monocyte activation, and expression of adherence and chemokine receptors indicative of altered migratory capacity, were also higher at 1–3 months post-infection in individuals who had mild SARS-CoV-2, but these were no longer elevated by 6–9 months post-infection. Perhaps most surprisingly, significantly more T-cells could be activated by polyclonal stimulation in individuals who had recently experienced a mild SARS-CoV-2, infection compared to individuals with other recent respiratory infections. These data are indicative of prolonged immune activation and systemic inflammation that persists for at least three months after mild or asymptomatic SARS-CoV-2 infections.

## 1. Introduction

Infection with SARS-CoV-2 has unusual effects on circulating immune cells. In general, respiratory infections are associated with rapid and sustained release of neutrophils and monocytes from the bone marrow, with no immediate change in production or function of lymphocytes [1]. In contrast, SARS-CoV-2 infections are consistently associated with lymphopenia, which is a reliable predictor of mortality [2]. The loss of T-cells may be due to apoptosis in the secondary lymphoid organs, or possibly inappropriate recruitment to the lungs and other organs [3,4]. Severe cases of SARS-CoV-2 infection are also associated with increased granularity and changes in nuclear morphology in neutrophils and monocytes, which are generally due to premature egress from the bone marrow and stunted maturation [5]. Whether myelopoiesis and egress are affected in mild COVID is not yet known.

Hospitalization with any respiratory infection is associated with long-term health consequences [6]; however, the consequences of COVID-19 appear to be particularly broad and include “long COVID” (a constellation of symptoms that can include severe fatigue often exacerbated by exercise, pain, and respiratory complications) [7,8], and a higher than expected risk of re-hospitalization for a variety of causes [8,9,10]. Although it has been documented that there are differences in acute immune responses in asymptomatic, mild, moderate, and severe cases of SARS-CoV-2 infection [11,12], it is not clear whether there is any risk of long-term immune dysregulation or health consequences in survivors of asymptomatic or mild cases. 

In this study we investigated whether there are any changes in phenotype and frequency of circulating leukocytes in individuals who had experienced mild COVID-19 (i.e., did not require medical attention), and compared them to those who had other mild respiratory infections. We found evidence of transient changes in circulating NK cells, T-cells, and monocytes 1–3 months post mild COVID-19, but not after other mild respiratory infections. These changes generally resolved 6–9 months post-COVID-19 infection, indicating that there is an early period of immune dysregulation greater than that of other respiratory infections, which occurs even after mild SARS-CoV-2 infection. 

## 2. Materials and Methods

### 2.1. Participant Recruitment and Blood Collection

Research participants with symptoms consistent with COVID-19 (as summarized in Table 1) were recruited from the greater Hamilton area (Ontario, Canada) between February 2020 and January 2021. All protocols were approved by the Hamilton Research Ethics Board (#10757 and #11471), and all participants provided informed consent. Venous blood was drawn in anti-coagulant-free vacutainers for the isolation of serum, and in heparin-coated vacutainers for experiments that required viable leukocytes. Serum and leukocytes were isolated as per standard protocols [13]. Immunophenotyping was performed on fresh blood within four hours of collection. None of the participants had been vaccinated against SARS-CoV-2 at the time of sample collection. Three participants who had a positive polymerase chain reaction (PCR) test for SARS-CoV-2 were either asymptomatic or had mild symptoms (i.e., headache or tiredness that lasted for a day). The remaining participants had symptoms that they reported as consistent with a typical respiratory infection (e.g., cough, fever, chills), but these were generally resolved within 2–4 weeks. The duration of symptoms and demographics of the 38 participants in this study (i.e., sex, age, body mass index (BMI), comorbid conditions, medications) are summarized in Table 1. Participants were characterized as having had COVID-19, ‘other respiratory infection’, or ‘indeterminate’, as detailed in Supplementary Table S1. 

Most participants did not consult a medical professional about their condition, consistent with the public health advice in the early months of the pandemic that individuals who did not need medical attention should stay home, and since none were hospitalized, we defined these as ‘mild’ infections. Participants that had recovered from COVID-19 gave blood at either 1–3 months post-symptom onset or 6–9 months post-symptom onset, but no participant gave blood at both time points. Therefore, there were no repeat measures from the same participant. 

### 2.2. Measurements of Anti-SARS-CoV-2 Antibodies

Anti-SARS-CoV-2 full-length spike (S) protein and receptor binding domain (RBD) IgG and IgA seropositivity were identified via validated serology enzyme-linked immunosorbent assay (ELISA), as described by Huynh et al., 2021 [14]. Briefly, NUNC™ Maxisorp 384 well plates (Thermo Fisher Scientific, Waltham, MA, USA) were coated with S (5 μg/mL) or RBD (2 μg/mL) antigens in 50 mM carbonate buffer (pH 9.6) overnight at 4 °C, and blocked with 3% skim milk in PBS-0.05% Tween-20. After washing with phosphate-buffered saline (PBS), plates were coated with diluted serum (1:100) for 1 h at room temperature, washed, and incubated with 25 μL alkaline phosphatase-conjugated antibodies goat anti-human IgG (1:2000) or goat anti-human IgA (1:500) (Jackson ImmunoResearch, West Grove, PA, USA). Plates were washed, and antibody levels were quantified by adding 50 μL of substrate buffer (0.27 µM p-nitrophenyl phosphate/diethanolamine buffer, 1 M, pH 9.6) and reading optical density (OD) at 405 nm detection every 1 min, with 490 nm reference on a BioTek 800TS microplate reader (BioTek Instruments Inc., Winooski, WT, USA).

### 2.3. Assessment of T Cell Activation Induced Markers (AIM) for SARS-CoV-2 Peptides

To detect antigen-specific T-cell recall responses, 100 µL of heparinized venous blood was cultured with an equal amount of Iscove’s Modified Dulbecco’s Medium, GlutaMAX™ Supplement (Invitrogen Life Technologies, Carlsbad, CA, USA) and antigens for 44 h in 96-well flat bottom plates at 37 °C. Wells were stimulated using SARS-CoV-2 peptide pools (PepTivator^®^ products from Miltenyi Biotec, Bergisch Gladbach, Germany) containing overlapping peptides covering the complete sequence of the membrane glycoprotein (M; #130-126-702), the nucleocapsid phosphoprotein (N; # 130-126-699), or the immunodominant sequence domains of the spike glycoprotein (S; #130-126-701); each used 1 μg/mL. Unstimulated wells served as negative controls, and polyclonal stimulation with CytoStim™ (0.5 µL/well, Miltenyi Biotec) was included as positive control. The fluorescently conjugated monoclonal antibody panel used for analysis is within Supplementary Table S2. Samples were run on a CytoFLEX LX (4 laser, Beckman Coulter, Brea, CA, USA). In this activation-induced marker assay, the antigen-specific T cells (AIM-positive) are defined by co-expression of CD25 and CD134 (OX40) for CD4^+^ T cells [15,16,17], and by co-expression of CD69 and CD137 (4-1BB) for CD8^+^ T-cells [18]. A positive assay result was defined as >3 standard deviations above an unstimulated sample (negative control well), consisting of at least 20 events. 

### 2.4. Quantification of Peripheral Immunophenotype

Circulating neutrophils, monocytes, T, B, and NK cells were quantitated by multi-color flow cytometry, as previously described [13,19]. Direct application of monoclonal antibodies (specificities outlined in Supplementary Table S2) to 100 µL of whole blood was performed for 30 min at room temperature. Following staining, samples were incubated with either 1 × Fix/Lyse Buffer (eBioscience, Thermo Fisher Scientific, Waltham, MA, USA) for 10 min, or following standard protocols for the FOXP3 Transcription Factor Staining Kit (eBioscience, Thermo Fisher Scientific, Waltham, MA, USA), washed with PBS, and resuspended in FACS Wash (5 mM EDTA, 0.5% BSA in PBS) for analysis with a CytoFLEX LX (4 laser, Beckman Coulter). Absolute counts for circulating immune populations were determined using CountBright™ absolute counting beads (Invitrogen Life Technologies, Carlsbad, CA, USA). Gating strategies and representative FACS dot plots to determine circulating immune populations are shown in Supplementary Figure S1. 

### 2.5. Measurements of Cytokines and C-Reactive Protein 

Serum cytokines interleukin-6 (IL-6), tumor necrosis factor (TNF), and C-reactive protein (CRP) were measured using the Ella™ Automated Immunoassay System (Bio-Techne, Minneapolis, MN, USA). Serum was diluted 1:2, as per the manufacturer’s protocol. The lower limits of quantification for CRP, IL-6, and TNF are 32 pg/mL, 0.28 pg/mL, and 0.3 pg/mL, respectively.

### 2.6. Statistical Analysis

Data and statistical analyses were done in FlowJo™ (Version 10.7.1 Ashland, OR, USA: Becton, Dickinson and Company; 2021), GraphPad Prism version 9 (San Diego, CA, USA), and R open-source software. Multiple group comparisons were tested using Welch’s One-Way ANOVA and the Games–Howell post-hoc test. As data were non-normally distributed, correlations were tested using the Spearman correlation. Outliers were removed using Grubbs’ test (α = 0.05).

## 3. Results

### 3.1. Demographics and Symptoms of Individuals Who Had SARS-CoV-2 and Other Respiratory Infections

During the early phase of the COVID-19 pandemic (February–June 2020), diagnostic PCR testing in Ontario was generally limited to individuals who were hospitalized, had a confirmed exposure with an infected person, or had travelled to an area with active infections. At the time, public health advice was that people who did not need medical attention should self-isolate at home without being tested. Serologic studies have been used to estimate an infection rate of 0.5–1.5% during the March-June ‘first wave’ [20]. Compounding the lack of testing were the high rates of influenza and unusually high rates of respiratory syncytial virus (RSV) infections [21]. Consequently, some people with respiratory infections in this early period were told by a health care professional that they had COVID-19 without a nasopharyngeal swab PCR test, and not all of our study participants with symptoms of mild respiratory illness had COVID-19 (Table 1). Participants were classified as having had COVID-19 if they received a positive diagnostic PCR test and/or had anti-SARS-CoV-2 antibodies (Supplementary Table S1). There were no statistically significant differences in age, sex, BMI, co-morbidities, medications, or duration of symptoms between individuals that had mild COVID-19 (*n* = 22) and individuals that had other mild respiratory infections (*n* = 11, Table 1). 

### 3.2. Survivors of Mild SARS-CoV-2 Infections Have Reduced T Cell Responses to SARS-CoV-2 Peptides 6–9 Months Post Symptom Onset

**Figure 1:**
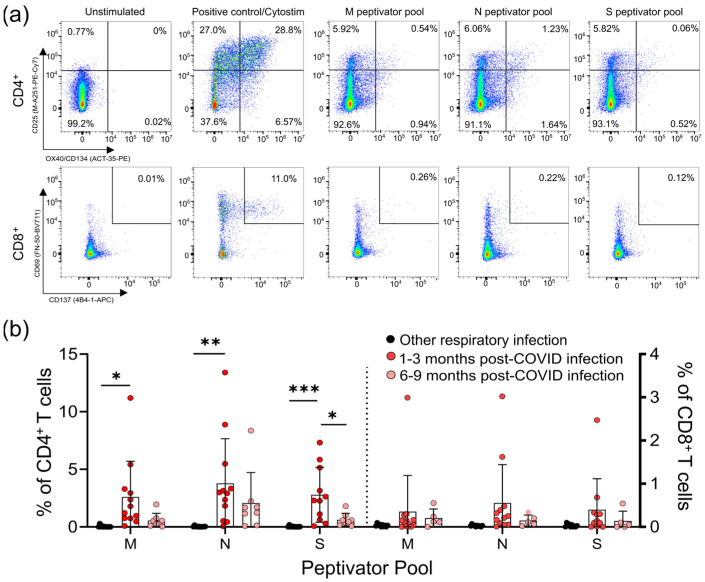
CD4^+^ and CD8^+^ T-cell responses to the M, N, and S peptide pools after mild SARS-CoV-2 infection. (**a**) The number of SARS-CoV-2-specific T-cells is measured as a percent of CD4^+^ T-cells expressing both CD25 and OX40, or CD8^+^ T-cells expressing both CD69 and CD137, after activation with the S, M, or N peptide pools 1–3 months and 6–9 months after infection. The polyclonal activator Cytostim is used as a positive control. (**b**) All COVID-19 seropositive participants had an increase in CD25^+^OX40^+^CD4^+^ T-cells in response to at least one of the M, N, or S antigens 1–3 months after mild COVID-19 infection, compared to seronegative individuals recovered from other mild respiratory infections. Each participant is indicated by a single data point: other respiratory infection *n* = 11; 1–3 months post COVID-19 infection *n* = 11; 6–9 months post COVID-19 infection *n* = 8. Multiple group comparisons were tested using Welch’s One-Way ANOVA and the Games–Howell post-hoc test; bars represent the mean ± standard deviation. * *p* < 0.05; ** *p* < 0.01; *** *p* < 0.001.

Lymphopenia is commonly reported in severe SARS-CoV-2 infections; however, it is not clear whether mild infections impact T-cell numbers or function. We used a flow cytometric activation-induced marker assay to measure T-cell responses to the SARS-CoV-2 structural membrane protein (M), nucleocapsid protein (N), and immunodominant regions of the spike (S) protein in all participants (Figure 1a). After 44 h incubation with antigen, this assay identifies antigen-specific CD4^+^ T-cells by induced co-expression of CD25 and CD134 (OX40), and antigen-specific CD8^+^ T-cells by co-expression of CD69 and CD137 (4-1BB) (Figure 1a) [15,16]. We identified five individuals who had mild respiratory symptoms, did not have a positive diagnostic PCR test, and were seronegative for SARS-CoV-2 antibodies, but had detectable levels of SARS-CoV-2 reactive T-cells to some, but not all, of the SARS-CoV-2 M, N, and S peptides (Supplementary Table S1). Consequently, we classified these five individuals as “indeterminate”, and did not include them in subsequent analyses (their demographic data is summarized in Table 1).

For analysis of the T-cell responses, we split our cohort into those without antibody or T -cell responses to SARS-CoV-2 (other respiratory infection; *n* = 11), and those with antibody responses to SARS-CoV-2 (COVID-19; *n* = 22), the majority of whom also had detectable T-cell responses to SARS-CoV-2 M, N, and S antigens (90.9%; *n* = 20/22) (Figure 1; Supplementary Figure S1). Of the 22 participants who had COVID-19 infections, 14 provided samples 1–3 months post-symptom onset, and 8 provided samples 6–9 months post-symptom onset. Consistent with other reports of lasting memory T-cell responses to SARS-CoV-2 infection [11], we observed that all COVID-19 participants that provided samples at 1–3 months or 6–9 months post-symptom onset had generated T-cell memory to at least one of the M, N, and S peptides pools (Figure 1b, Supplementary Table S3). 

We saw similar frequencies of CD4^+^ T-cell responses to all SARS-CoV-2 antigens, and these were highest in COVID-19 patients 1–3 months post symptom onset (means of ~3–4% of CD4^+^ T-cells), and were significantly higher than in individuals with other respiratory infections (Figure 1b). Compared to COVID-19 patients 1–3 months post-symptom onset, responses were reduced in COVID-19 patients 6–9 months post-symptom onset, and were not significantly different to those in individuals recovered from other respiratory infections. Similar results were found for CD8^+^ T-cell responses, being highest in COVID-19 patients 1–3 months post-symptom onset (means of ~0.4–0.5% of CD8^+^ T-cells), although there were no significant differences between the groups. Together, these data indicate there are long-term differences in T-cell functionality in COVID-19 survivors.

### 3.3. Survivors of Mild SARS-CoV-2 Infections Have Evidence of Sustained Inflammation 1–3 Months Post Symptom Onset

**Figure 2:**
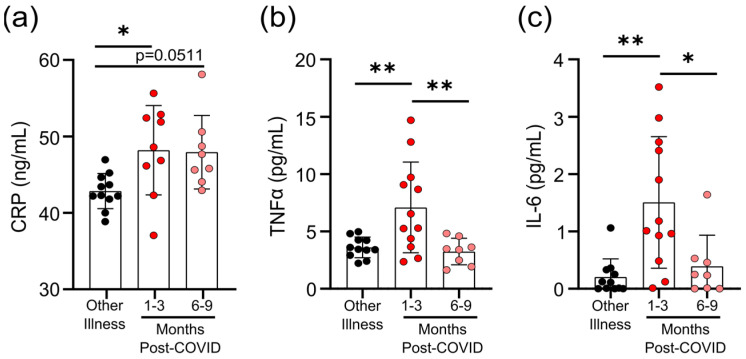
Transient increases in soluble mediators of inflammation occur after mild SARS-CoV-2 infection. (**a**) CRP levels in serum were higher 1–3 months after mild COVID-19 infection, and there was a trend towards remaining elevated 6–9 months after mild COVID-19 infection, compared to levels observed in seronegative individuals after other respiratory infections. Serum TNF levels (**b**) and IL-6 levels (**c**) were higher 1–3 months after COVID-19 infection, but returned to levels seen in individuals who had other respiratory infections by 6–9 months. Each participant is indicated by a single data point: other respiratory infection *n* = 11; 1–3 months post-COVID *n* = 9–12; 6–9 months post-COVID *n* = 8. Multiple group comparisons were tested using Welch’s One-Way ANOVA and the Games–Howell post-hoc test; bars are presented as mean ± standard deviation. * *p* < 0.05; ** *p* < 0.01.

Prolonged immune activation can occur after recovery from severe infections, and is thought to contribute to malaise and other symptoms in survivors [22]. One of the unusual features of COVID-19 is that a significant number of patients with mild to moderate illness report symptoms weeks to months after infection, despite having cleared the virus [23]. Although the participants in our study generally had symptoms resolve within 1 month post-infection (Table 1), we have nonetheless found evidence of prolonged inflammation. Seropositive individuals had higher levels of CRP, TNF, and IL-6 in circulation 1–3 months post-symptom onset; but in samples from individuals 6–9 months post-symptom onset, levels were equivalent to those individuals who were seronegative and were recovered from other respiratory infections (Figure 2a–c). 

**Figure 3:**
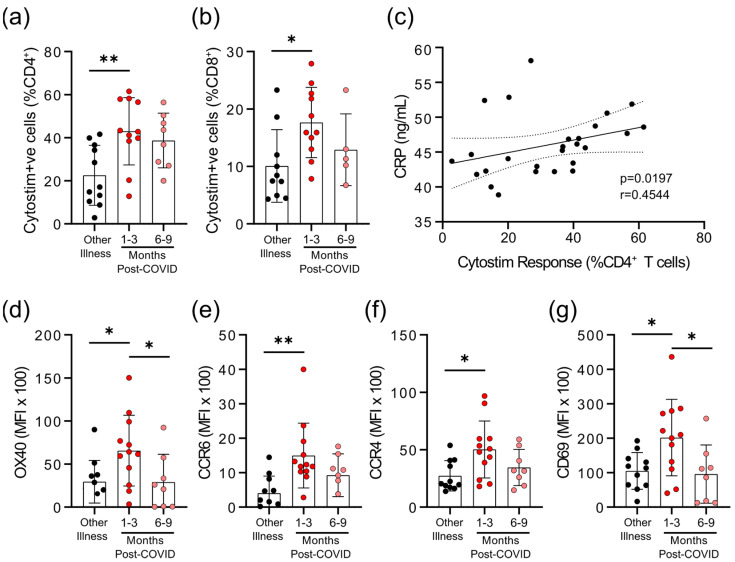
Evidence of prolonged T-cell activation after mild SARS-CoV-2 infection. The polyclonal activator Cytostim was used to measure T-cell responses. COVID-19 seropositive individuals had a higher proportion of CD4^+^ (**a**) and CD8^+^ (**b**) activated T-cells 1 to 3 months after infection, compared to seronegative individuals after other respiratory infections. (**c**) The number of CD4^+^ T-cells that responded to polyclonal stimulation correlated with CRP in COVID-19 seropositive individuals. (**d**–**g**) Activation markers OX40, CCR6, CCR4, and CD69 on CD4^+^ T-cells were higher 1–3 months after COVID-19 infection, compared to after other respiratory infections, after Cytostim exposure. Each participant is indicated by a single data point: other respiratory infection *n* = 7–11; 1–3 months post COVID-19 infection *n* = 11–12; 6–9 months post COVID-19 infection *n* = 5–8. Multiple group comparisons in (**a**,**b**) and (**d**–**g**) were tested using Welch’s One-Way ANOVA and the Games–Howell post-hoc test; bars are presented as mean ± standard deviation. Data in C was assessed by Spearman’s rank correlation. * *p* < 0.05; ** *p* < 0.01.

We observed that there were differences in the degree of epitope independent polyclonal activation of T-cells between the COVID-19 and non-COVID-19 groups. When whole blood was stimulated with Cytostim, which acts as a superantigen by crosslinking the T-cell receptor (TCR) (regardless of TCR variable β gene usage) and major histocompatibility complex (MHC), there was a statistically significant difference in the percent of CD25^+^OX40^+^CD4^+^ T-cells and CD69^+^CD137^+^CD8^+^ T-cells in COVID-19 patients 1–3 months post-symptom onset (Figure 3a,b). Whether this superantigen-like crosslinking activation contributes to, or is a result of, systemic inflammation is not clear, but CRP levels correlated with the percent of Cytostim-activated CD4^+^ T-cells (Figure 3c). CD4^+^ T-cells in the COVID-19 group also had higher expression levels of the activation markers OX40 and CD69, as well as the chemokine receptors CCR6 and CCR4, than the other respiratory infection group, implying a stronger activation (Figure 3d; Supplementary Figure S2). Although the levels of OX40 and CD69 were reduced in samples collected 6–9 months post-symptom onset compared to samples collected 1–3 months post-symptom onset, levels of CCR4 and CCR6 remained similar. Collectively, these data imply that there may be subtle changes in inducible T-cell activation in convalescent SARS-CoV-2 patients that do not occur in response to other respiratory infections. 

### 3.4. Survivors of Mild SARS-CoV-2 Infections Have Changes in Circulating Immunophenotype 1–3 Months Post-Symptom Onset

**Figure 4:**
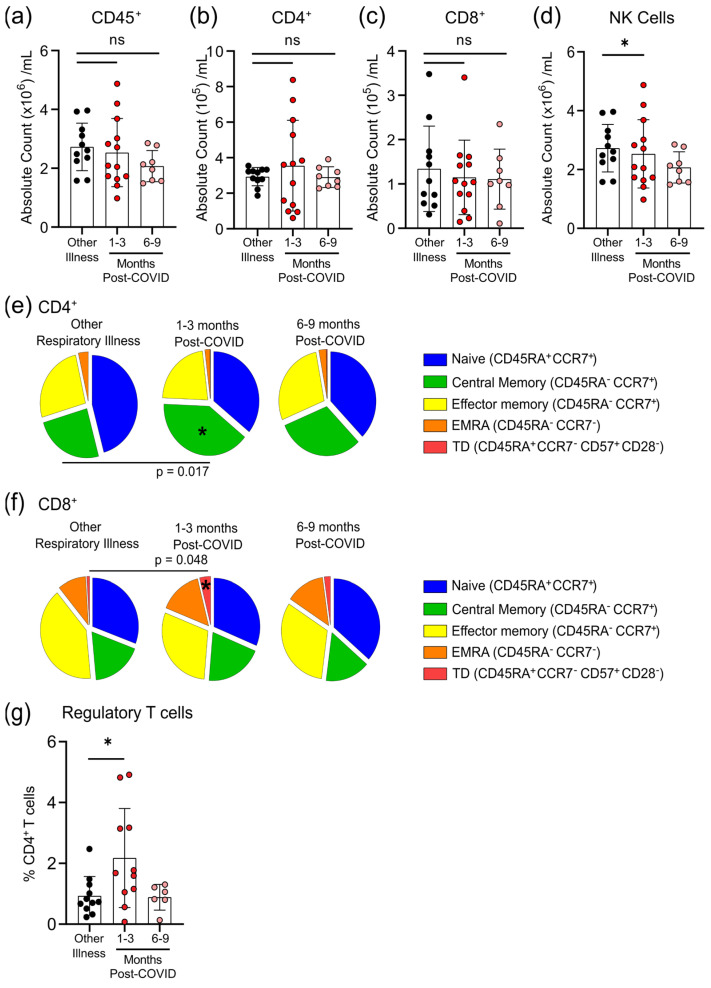
Transient changes in circulating lymphocytes occur 1–3 months after COVID-19 infection. Absolute numbers of circulating CD45^+^ T-cells (**a**), CD4^+^ T-cells (**b**), and CD8^+^ T-cells (**c**) were not different after 1–3 months or 6–9 months post-symptom presentation in seropositive individuals recovered from mild COVID-19 infection, compared to seronegative individuals recovered from other respiratory infections; however, NK cell numbers (**d**) were lower in the 1–3 months post-COVID-19 infection group. At 1–3 months post recovery from COVID-19 there was an increase in CD45RA^−^CCR7^+^ central memory CD4^+^ T-cells (**e**), and an increase in CD45RA^+^CCR7^−^CD57^+^CD28^−^ terminally differentiated CD8^+^ T-cells (**f**), compared to individuals recovered from other respiratory infections, but these differences were not apparent in individuals who had recovered from COVID-19 6–9 months prior. (**g**) Levels of circulating regulatory T-cells (measured as a % of CD4^+^ T-cells) were higher in individuals 1–3 months post COVID-19 infection. Each participant is indicated by a single data point: other respiratory infection *n* = 11; 1–3 months post COVID-19 infection *n* = 11–13; 6–9 months post COVID-19 infection *n* = 8. ns—not statistically significant. Multiple group comparisons were tested using Welch’s One-Way ANOVA and the Games–Howell post-hoc test; bars are presented as mean ± standard deviation. * *p* < 0.05.

The total numbers of circulating CD45^+^ cells, CD4^+^ T-cells and CD8^+^ T-cells were not different between individuals who had other respiratory infections and those who had COVID-19 at either 1–3 months or 6–9 months post-symptom onset (Figure 4a–c). Compared to participants who were recovered from other respiratory infections, there was an expansion of regulatory T-cells (T_regs,_ defined as CD45^+^CD3^+^CD4^+^CD25^+^CD127^low^FOXP3^+^) and a decrease in natural killer (NK) cells (CD45^+^CD56^+^/NKp46^+^) in COVID-19 patients at 1–3 months post-symptom onset that returned by 6–9 months post-symptom onset to levels observed in individuals with non-COVID-19 respiratory infections (Figure 4d,g). We also measured the proportions of circulating CD4^+^ and CD8^+^ T-cells that were naïve cells (CD45RA^+^CCR7^+^), central memory cells (CD45RA^−^CCR7^+^), effector memory cells (CD45RA^−^CCR7^−^), terminally differentiated effector memory cells re-expressing CD45RA (T_EMRA_; CD45RA^+^CCR7^−^), and terminally differentiated cells (CD45RA^+^CCR7^−^CD57^+^CD28^−^). Compared to patients with non-COVID-19 respiratory infections, there was an expansion of central memory CD4^+^ T-cells and terminally differentiated CD8^+^ T-cells in COVID-19 patients at 1–3 months post-symptom onset that returned to levels observed in patients with other respiratory infections by 6–9 months post-symptom onset (Figure 4e,f). There were no significant differences between participants who had other respiratory infections and those who had COVID-19 for any other measured CD4^+^ and CD8^+^ T-cell subsets. 

**Figure 5:**
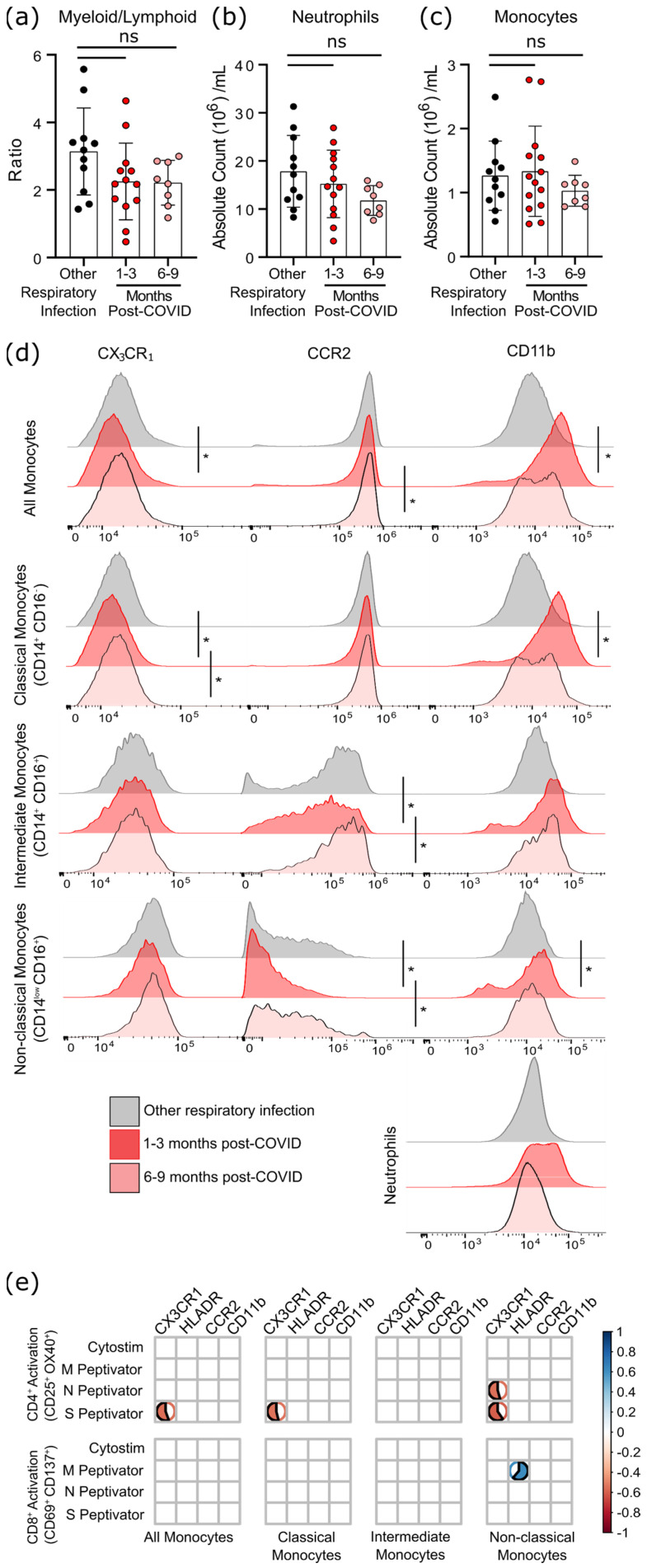
Evidence of sustained cellular inflammation after mild SARS-CoV-2 infection. (**a**) There was a trend towards a decreasing ratio of myeloid to lymphoid cells after SARS-CoV-2 infection, compared to individuals recovered from other infections, which was driven by a decrease in circulating neutrophils (**b**). Although total monocyte numbers did not change after infection (**c**), surface expression of the migratory markers CX_3_CR_1_ and CCR2 decreased transiently (**d**). Concurrent increases in surface expression of the migration and activation marker CD11b implies that the monocytes were activated. (**e**) Correlation analysis (Spearman’s correlation) of SARS-CoV-2 specific CD4^+^ and CD8^+^ T-cell responses with measures of monocyte activation and migratory potential. Multiple group comparisons in (**a**–**d**) were tested using Welch’s One-Way ANOVA and the Games–Howell post-hoc test; in (**a**–**c**) bars are presented as mean ± standard deviation, and each dot indicates a participant. The spread of expression of monocyte surface markers in d was visualized by concatenating uncompensated CD45^+^CD19^−^CD3^−^CD56^−^CD11b^+^HLADR^+^CD14^+^ events in FlowJo for each infection group prior to overlaying geometric mean fluorescence intensity expression data from all participants onto the same histogram plot. Other respiratory infection (grey) *n* = 11, 1–3 months after COVID-19 infection (red) *n* = 14, 6–9 months from COVID-19 infection (pink) *n* = 8. ns—not statistically significant. Data in (**e**) were assessed with the rcorr function in the Hmisc package in R, and only statistically significant associations are shown. * *p* < 0.05.

Although six months after SARS-CoV-2 infection there was a statistical trend towards a lower ratio of myeloid to lymphoid cells, this was due to a non-significant decrease in neutrophil numbers rather than changes in the number of monocytes (Figure 5a–c). However, as circulating monocytes are a sensitive marker of chronic inflammation, we assessed monocyte subsets, as well as their expression of migratory and activation markers (Figure 5d, Supplementary Tables S4 and S5). Classical monocytes (CD14^+^CD16^−^) expressing CCR2 are the first to leave the bone marrow. They have a half-life in the circulation of less than 24 h, since they are either recruited to sites of acute inflammation in response to CCL2/monocyte chemoattractant protein-1 (MCP-1), or differentiate into CX_3_CR_1_-expressing intermediate (CD14^+^CD16^+^) monocytes [24,25]. Classical monocytes increase in the circulation during acute infection so, as expected, there was no difference in the number of circulating monocytes after COVID-19 infection had resolved (Figure 5c). At 1–3 months after infection, levels of CCR2 were lower on intermediate and non-classical monocyte populations (Figure 5d), implying that cells with the highest levels of CCR2 emigrated from the circulation. 

In general, the chemokine receptor CX_3_CR_1_ is expressed more highly on intermediate and non-classical (CD14^low^CD16^+^) monocytes, and is associated with recruitment to the tissues or vasculature and repair of damage in response to CX_3_CL_1_/fractalkine [26,27]. A transient decrease in CX_3_CR_1_-expressing classical monocytes was found 1–3 months post COVID-19 infection, which likely indicates that monocytes with the highest levels of CX_3_CR_1_ (and therefore the most responsive to CX_3_CL_1_) had emigrated from the circulation. Expression of the integrin CD11b was also transiently increased on monocytes at 1–3 months post-symptom onset, but not neutrophils, providing further evidence of transient but systemic immune activation, and possible changes in the migratory capacity of monocytes after mild SARS-CoV-2 infection. 

Changes in monocyte activation markers could be due to the increase in basal inflammation, or could be proportionate to the SARS-CoV-2 specific immune response. We found that decreasing CX_3_CR_1_ expression was most strongly associated with the percentage of S-antigen specific CD4^+^ T-cells (*p* = 0.0389), and that there was a strong association between M-antigen specific CD8^+^ T-cells and human leukocyte antigen-DR isotype (HLA-DR) expression on non-classical/patrolling monocytes (*p* = 0.0198) (Figure 5e). There was no relationship between polyclonal T-cell activation (i.e., Cytostim) and myeloid activation, implying that these changes are not due to the general increase in T-cell activation observed in convalescent patients, but rather SARS-CoV-2 specific responses. 

## 4. Discussion

Infections severe enough to require hospitalization can have long-term health consequences, but one of the unusual features of COVID-19 is that a significant proportion of mild infections have lasting cardiovascular, respiratory, and neurologic consequences [23,28]. The range of long-term health consequences and the multi-organ immune pathology observed post-SARS-CoV-2 infection implies that there may be a greater degree of immune dysregulation than that commonly observed after infection with other respiratory pathogens. Importantly, the participants in this study did not have “long-COVID” in that they reported that their symptoms were mild, and had mostly resolved within 2–4 weeks (Table 1); however, participants with even mild COVID-19 symptoms experienced elevated inflammation and immune activation that endured at least 1–3 months after their infection had resolved. The fact that these participants had increased measures of systemic inflammation (e.g., CRP), and lasting phenotypic and functional changes to both monocytes and T-cells, implies that inflammatory responses to mild COVID-19 infections are more protracted than expected. 

The proportions of memory CD4^+^ and CD8^+^ T-cells specific for SARS-CoV-2 S, M, and N proteins we observed were consistent with those reported for convalescent patients who had had more severe infections, implying that disease severity is not proportionate to SARS-CoV-2 memory T-cell responses [29,30]. We also detected SARS-CoV-2 reactive T-cells for *n* = 5 individuals who had mild respiratory symptoms that did not have a positive diagnostic PCR test, and were seronegative for anti-SARS-CoV-2 antibodies. Although it is possible these individuals had been infected with SARS-CoV-2 and had antibodies at some point, these data are also consistent with reports of pre-existing cross-reactive T-cells to SARS-CoV-2 antigens in as many as 30% of individuals [29]. Previous studies have reported differences in the expression of activation markers on SARS-CoV-2 specific memory CD4^+^ T-cells between exposed but SARS-CoV-2 seronegative individuals, compared to those who had either mild or severe infections [11]; however, to our knowledge, this is the first report of elevated activation of T-cells by MHC/TCR crosslinking (i.e., Cytostim) in convalescent COVID-19 patients. Cytostim-treated CD4^+^ T-cells in participants with mild COVID-19 1–3 months post-symptom onset, compared to those in participants recovered from other respiratory infections, had higher expression of the activation markers OX40, CCR4, CD69, and CCR6, and OX40 and CD69 expression remained elevated after 6–9 months post-symptom onset. There were some differences between participant groups in the expression of activation markers on circulating unstimulated cells, demonstrating that these differences are not restricted to polyclonally activated cells. There are several possible explanations for this result. CD4^+^ T-cells from 1–3 months post-symptom onset of COVID-19 may be primed to respond quicker and more strongly to activation signals. Alternatively, since the number of activated CD4^+^ T-cells in this participant group correlated strongly with rising CRP levels, it is also possible that there may be circulating cytokines (e.g., IL-6) or other factors that lower the threshold for T-cell activation.

Our observations of transient increases in central memory CD4^+^ T-cells and terminally differentiated CD8^+^ T-cells are consistent with T-cell responses to acute viral infection [31,32], and with previous observations that survivors of mild SARS-CoV-2 infections have lasting memory responses [11]; however, a pronounced increase in regulatory T-cells is generally associated with minimizing pathology during the acute, not the convalescent, phase of respiratory viral infections [31,32]. Severe SARS-CoV-2 infections are associated with the development of autoantibodies that contribute to severity [33]. Mild SARS-CoV-2 infections are associated with autoimmune inflammatory syndromes (e.g., arthritis, vasculitis) after the primary infection has resolved [34,35]. Whether this expansion of T_regs_ protects from autoimmune sequelae is not clear, but this has been reported to occur after other zoonotic infections [36]. Appropriate regulation of T_regs_ may be especially important in SARS-CoV-2 infections, since pathology of these infections are caused in part by dysregulation of transforming growth factor-β production, a major cytokine produced by T_regs_ [37] that is required for their appropriate differentiation [38]. 

Severe COVID-19 infections are associated with a dysregulation of myelopoiesis that is so extreme that monocytes and neutrophils are unrecognizable by blood smear [39], and have dramatic changes in granularity, size, and surface marker expression [40]. Both mild and severe COVID-19 are associated with changes in the number and migratory potential of dendritic cells for at least 7 months post-infection [41]. We do not know if the transient changes in expression of CCR2 and CX_3_CR_1_ on circulating monocytes 1–3 months after infection are due to emigration of cells with the highest expression of those markers to inflamed or damaged tissues; however, CCL2 has been implicated in recruitment of monocyte to the lungs during infection [42]. The observation that decreasing CX_3_CR_1_ is associated with the level of SARS-CoV-2-specific T-cells may imply that there is a relationship between immune responsiveness and innate immune activation. There was no relationship between polyclonal T-cell activation (i.e., Cytostim) and myeloid activation, implying that this relationship is not due to the general increase in T-cell activation observed in convalescent patients, but rather SARS-CoV-2-specific responses. Furthermore, we observed prolonged elevation of cytokines, perhaps a lasting effect of the cytokine storm present in many COVID-19 patients, even in mild infections [43,44]. These observations in combination with the elevated expression of monocyte CD11b, which increases during acute and chronic inflammation and alters monocyte migration and adherence to the vasculature [45], imply even though the symptoms of mild COVID-19 infection may resolve in weeks, immune activation persists for at least 1–3 months.

Our study has identified key differences between immune responses following SARS-CoV-2 infection and other respiratory infections, although a limitation was that we were unable to determine the type(s) of non-COVID-19 respiratory infections. All participants in the study were ill before SARS-CoV-2 vaccines were available, and were unvaccinated at the time of sample collection. Although we identify some intriguing differences at distinct time points post-infection, this was not a longitudinal study, so different individuals were compared in the 1–3-month and 6–9-month post-infection groups. It will be interesting to compare our data on immune responses generated by primary infection to future studies examining post-infection immune activation in vaccinated individuals with mild breakthrough infections. 

Collectively, these data provide evidence that mild, and, in some individuals, even asymptomatic SARS-CoV-2 infections can lead to sustained immune activation after resolution of symptoms, which is not observed in response to other mild respiratory infections. Whether this immune activation is more pronounced in patients with long-COVID, or more serious infections, remains to be seen.

## Figures and Tables

**Table 1:** Participant demographics ^1^.

	Other Respiratory Infections (Not COVID-19) *n* = 11	COVID-19 Infections *n* = 22	Indeterminate *n* = 5	*p* Value ^1^
Age (mean ± STDEV)	55 ± 16	55 ± 15	57 ± 11	0.9249
Sex (% female)	8 (73%)	11 (50%)	3 (60%)	0.2783
BMI (kg/m^2^)	24.0 ± 2.9	25.0 ± 3.6	24.5 ± 2.3	0.4172
**Health Conditions (frequency)**				
Asthma	1 (9%)	1 (5%)	1 (20%)	-
COPD (including emphysema and chronic bronchitis)	1 (9%)	0 (0%)	0 (0%)	-
Other lung disease	0 (0%)	1 (5%)	0 (0%)	-
Diabetes	0 (0%)	3 (14%)	0 (0%)	-
Hypertension	1 (9%)	1 (5%)	1 (20%)	-
Heart disease	0 (0%)	2 (9%)	0 (0%)	-
Cancer	2 (18%)	2 (9%)	0 (0%)	-
Autoimmune condition	1 (9%)	1 (5%)	1 (20%)	-
**Medications**				
Number of medications (mean ± STDEV)	1 ± 2	2 ± 2	1 ± 1	0.2049
**Symptoms (frequency)**				
Cough	6 (55%)	13 (59%)	2 (40%)	-
Shortness of breath	4 (36%)	9 (41%)	1 (20%)	-
Chest pain	3 (27%)	9 (41%)	2 (40%)	-
Fever	3 (27%)	12 (55%)	1 (20%)	-
Feeling generally unwell	9 (82%)	21 (95%)	2 (40%)	-
Abnormally tired	7 (64%)	20 (91%)	2 (40%)	-
New confusion	4 (36%)	1 (5%)	1 (20%)	-
New generalized muscle aches and pains	5 (45%)	16 (73%)	2 (40%)	-
New joint pain	3 (27%)	5 (23%)	2 (40%)	-
Earache/infection	0 (0%)	1 (5%)	0 (0%)	-
Headache	6 (55%)	14 (64%)	1 (20%)	-
Runny/stuffy nose	7 (64%)	7 (32%)	2 (40%)	-
Sinus pain	2 (18%)	4 (18%)	1 (20%)	-
Sore/scratchy throat	7 (64%)	10 (45%)	2 (40%)	-
Loss of appetite	4 (36%)	8 (36%)	2 (40%)	-
Loss of taste/smell	2 (18%)	11 (50%)	1 (20%)	-
Duration of Symptoms				
No symptoms—1 week	4 (36%)	3 (14%)	1 (20%)	-
2–4 weeks	3 (27%)	11 (50%)	4 (80%)	-
4 or more weeks	4 (36%)	8 (36%)	0	-
**Diagnosis**				
PCR test for SARS-CoV-2 performed	0 (0%)	15 (68%)	0 (0%)	-
Told by a health care professional that they had SARS-CoV-2/COVID-19 without a PCR test	1 (8%)	5 (25%)	0 (0%)	-

^1^ Statistical comparisons were made between ‘other respiratory infections’ and ‘COVID-19 infections’ groups only, since the ‘indeterminate’ group was not used in subsequent analyses. Student’s unpaired parametric *t*-test was used to compare age and BMI, which were normally distributed. Sex distribution was measured by Fisher’s exact test to analyze a 2 × 2 contingency table. Differences in the number of medications, which was not normally distributed, was measured using an unpaired non-parametric test not assuming Gaussian distribution.

## Data Availability

Data are available from the corresponding author upon reasonable request.

## Supplementary Materials

The following are available online at https://www.mdpi.com/article/10.3390/v13112239/s1, Figure S1: Gating strategies for flow cytometry data analysis, Figure S2: Expression of activation markers on unstimulated T cells, Table S1: Criteria and identification of individuals with SARS-CoV-2 and other respiratory infections, Table S2: Fluorophore-conjugated antibodies used for flow cytometry, Table S3: Summary of SARS-CoV-2 PepTivator AIM assay T-cell activation observations, Table S4: All immune parameter data analysis, Table S5: Monocyte surface receptor expression as mean fluorescence intensity (MFI).
