# Supplementary material for: Lasting Changes to Circulating Leukocytes in People with Mild SARS-CoV-2 Infections
Source: Viruses. 2021 Nov 8;13(11):2239. doi: 10.3390/v13112239 (PMC8622816; doi:10.3390/v13112239)
Supplement: Supplementary file 1 [file viruses-13-02239-s001.zip › viruses-1441062-supplementary Figure S1.pdf]

Supplementary Figure S1. Gating strategies for flow cytometry data analysis

A) Antigen Induced Memory (AIM) panel gating strategy

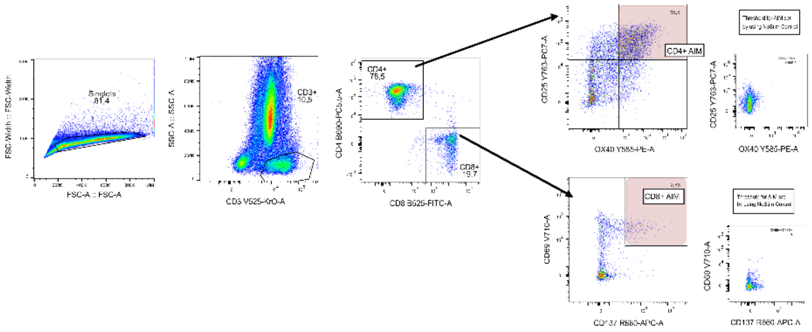

B) T cell phenotype panel gating strategy

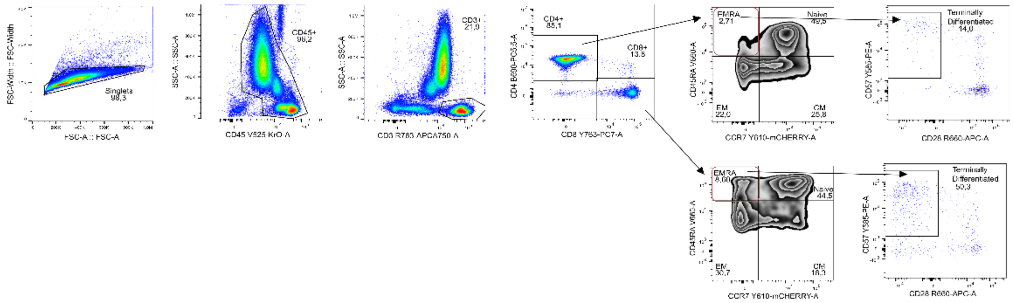

C) Lymphocyte panel gating strategy

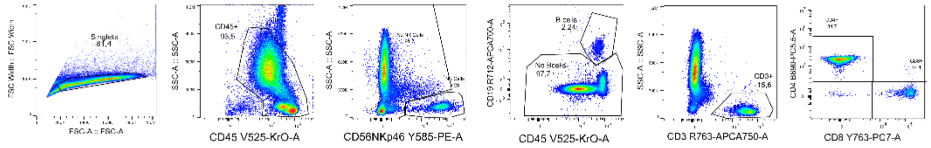

D) Myeloid panel gating strategy

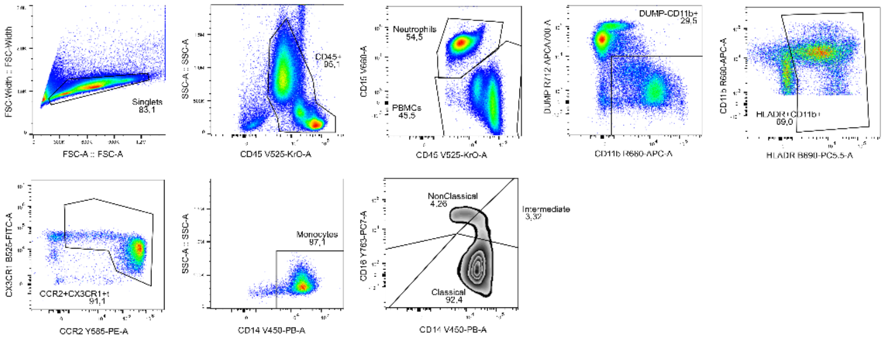

E) Regulatory T cell panel gating strategy

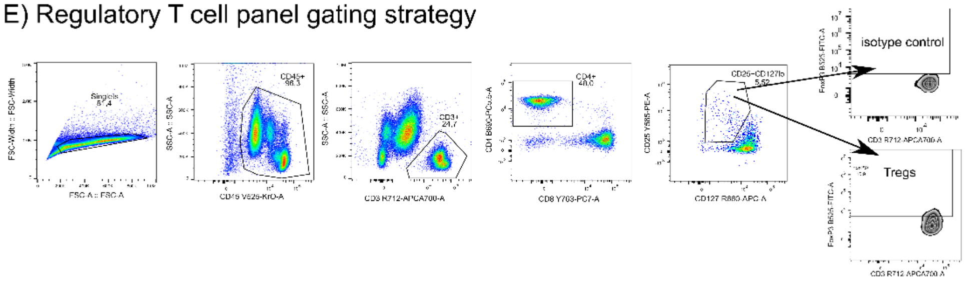

Fluorophores used, suppliers, and dilutions are described in Supplementary Table S2.
