# Supplementary material for: Lasting Changes to Circulating Leukocytes in People with Mild SARS-CoV-2 Infections
Source: Viruses. 2021 Nov 8;13(11):2239. doi: 10.3390/v13112239 (PMC8622816; doi:10.3390/v13112239)
Supplement: Supplementary file 1 [file viruses-13-02239-s001.zip › viruses-1441062-supplementary Figure S2.pdf]

Supplementary Figure S2. Expression of activation markers on unstimulated T cells

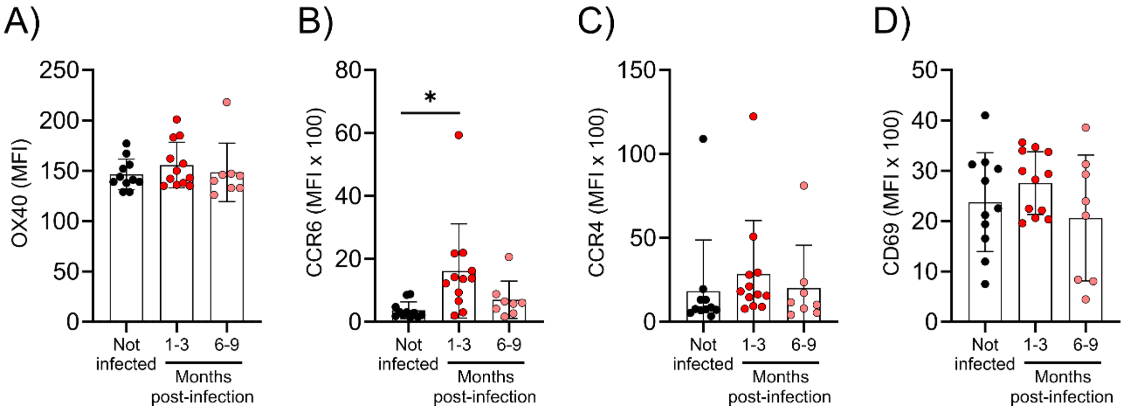

Expression of activation markers OX40, CCR6, CCR4, and CD69 on unstimulated $CD4^+$ T-cells from participants with other respiratory infections, 1–3 months after COVID-19 infection, and 6–9 months after COVID-19 infection. Each participant is indicated by a single data point: other respiratory infection n = 7–11; 1–3 months post COVID-19 infection n = 11–12; 6–9 months post COVID-19 infection n = 5–8. Multiple group comparisons were tested using Welch's One-Way ANOVA and the Games–Howell post-hoc test; bars are presented as mean ± standard deviation. * $p < 0.05$.
