# Supplementary material for: Lasting Changes to Circulating Leukocytes in People with Mild SARS-CoV-2 Infections
Source: Viruses. 2021 Nov 8;13(11):2239. doi: 10.3390/v13112239 (PMC8622816; doi:10.3390/v13112239)
Supplement: Supplementary file 1 [file viruses-13-02239-s001.zip › viruses-1441062-supplementary Table S1.pdf]

Supplementary Table S1. Criteria and identification of individuals with SARS-CoV-2 and other respiratory infections

			Serology *	
**SARS-CoV-2 Infection status**	**Diagnostic PCR Test**	**T cell response†**	**Negative**	**Positive**
**SARS-CoV-2 Infection (n = 22)**	Yes	Negative	0	1
		Positive	0	14
	No	Negative	0	1
		Positive	0	7
**Indeterminate (n = 5)**	Yes	Negative	0	0
		Positive	0	0
	No	Negative	0	0
		Positive	5	0
**Other respiratory infection (n = 11)**	Yes	Negative	0	0
		Positive	0	0
	No	Negative	11	0
		Positive	0	0

*A positive result for serology was defined as having either IgA or IgG to either the SARS-CoV-2 spike or S-RBD as in [15].

†A positive T cell response included evidence of either $CD4^+$ or $CD8^+$ T cell activation in response to peptides from the SARS-CoV-2 M, nucleocapsid or spike proteins.
