# Supplementary material for: Lasting Changes to Circulating Leukocytes in People with Mild SARS-CoV-2 Infections
Source: Viruses. 2021 Nov 8;13(11):2239. doi: 10.3390/v13112239 (PMC8622816; doi:10.3390/v13112239)
Supplement: Supplementary file 1 [file viruses-13-02239-s001.zip › viruses-1441062-supplementary Table S2.pdf]

Supplementary Table S2. Fluorophore-conjugated monoclonal antibodies used for flow cytometry

Cell Surface Marker	Fluorophore	Clone	Company	Cat No.	Antibody ID	Antibody Dilution
**AIMS activation assays**						
**CD3**	BV510	UCHT1	BD Biosciences	563109	AB_2732053	1/50
**CD4**	BB700	SK3	BD Biosciences	566392	AB_2744421	1/50
**CD25**	PECy7	M-A251	BD Biosciences	557741	AB_396847	1/50
**CD134 (OX40)**	PE	ACT-35	BioLegend	350004	AB_10645478	1/100
**CD39**	PEDazzle594	A1	BioLegend	328224	AB_2564319	1/25
**CD8**	BB515	RPA-T8	BD Biosciences	564526	AB_2744458	1/50
**CD137**	APC	4B4-1	BioLegend	309810	AB_830672	1/50
**CD69**	BV711	FN50	BioLegend	310944	AB_2566466	1/50
**T cell phenotype staining**						
**CD45**	BV510	HI30	BioLegend	304036	AB_2561940	1/100
**CD3**	APCeF780	UCHT1	eBioscience	47-0038-42	AB_1272042	1/50
**CD4**	PerCPCy5.5	OKT4	eBioscience	45-0048-42	AB_10804390	1/100
**CD8**	PECy7	RPA-T8	BD Pharmingen	557750	AB_396856	1/100
**CD15**	AF700	W6D3	BioLegend	323026	AB_2561427	1/100
**CD56**	AF700	5.1H11	BioLegend	362522	AB_2564099	1/50
**CD14**	AF700	M5E2	BioLegend	301822	AB_493747	1/100
**CD19**	AF700	HIB19	eBioscience	56-0199-42	AB_2043819	1/50
**CD45RA**	BV650	HI100	BioLegend	304136	AB_2563653	1/33
**CCR7**	PE-eFluor 610	3D12	eBioscience	61-1979-42	AB_2574592	1/20
**CD57**	PE	TB01	eBioscience	12-0577-42	AB_10804531	1/25
**CD28**	APC	CD28.2	eBioscience	17-0289-42	AB_10597439	1/50
**Lymphoid staining**						
**CD3**	APCeF780	UCHT1	eBioscience	47-0038-41	AB_1272042	1/200
**CD4**	PerCPCy5.5	OKT4	eBioscience	45-0048-42	AB_1272042	1/100
**CD8**	PE-Cy7	RPA-T8	BD Pharmingen	557750	AB_396856	1/50
**CD45**	BV510	HI30	BioLegend	304036	AB_2561940	1/100
**CD56**	PE	5.1H11	BioLegend	362524	AB_2564161	1/50
**NKp46**	PE	9-E2	BD Biosciences	557991	AB_396974	1/50
**CD19**	AF700	HIB19	eBioscience	56-0199-42	AB_2043819	1/50
**Myeloid staining**						
**CD45**	BV510	HI30	BioLegend	304036	AB_2561940	1/100
**CD16**	PE-Cy7	CB16	eBioscience	25-0168-42	AB_10714839	1/100
**CD14**	BV421	M5E2	BioLegend	301830	AB_10959324	1/100
**CCR2**	PE	K036C2	BioLegend	357205	AB_2562058	1/50
**CD11b**	APC	ICRF44	BD Biosciences	561015	AB_398456	1/50
**HLA-DR**	PerCPCy5.5	LN3	eBioscience	45-9956-42	AB_10718537	1/100
**CX3CR1**	FITC	2A9-1	Cedarlane	D070-4	NA	1/50
**CD15**	BV650	SSEA-1	BioLegend	323033	AB_2562499	1/200
**CD3**	AF700	UCHT1	BD Biosciences	557943	AB_396952	1/50
**CD56**	AF700	5.1H11	BioLegend	362522	AB_2564099	1/50
**CD19**	AF700	HIB19	eBioscience	56-0199-42	AB_2043819	1/50
**Regulatory T cell staining**						
**CD45**	BV510	HI30	BioLegend	304036	AB_2561940	1/100
**CD3**	AF700	UCHT1	BD Biosciences	557943	AB_396952	1/33
**CD4**	PerCPCy5.5	OKT4	eBioscience	45-0048-42	AB_1272042	1/100
**CD8**	PECy7	RPA-T8	BD Pharmingen	557750	AB_396856	1/100
**CD25**	PE	BC96	eBioscience	12-0259-41	AB_1659683	1/17
**CD127**	APC	eBioRDR5	eBioscience	17-1278-41	AB_1659673	1/20
**FoxP3**	FITC	236A/E7	eBioscience	11-4777-41	AB_11151147	1/10
