# Supplementary material for: Lasting Changes to Circulating Leukocytes in People with Mild SARS-CoV-2 Infections
Source: Viruses. 2021 Nov 8;13(11):2239. doi: 10.3390/v13112239 (PMC8622816; doi:10.3390/v13112239)
Supplement: Supplementary file 1 [file viruses-13-02239-s001.zip › viruses-1441062-supplementary Table S3.pdf]

Supplementary Table S3. Summary of SARS-CoV-2 peptivator AIM assay T cell activation observations

Time Since Infection	T cell population	Peptivator Pool (n and %)	
1-3 months from infection	CD4 (CD25+OX40+) N=12	M	12 (100%)
		N	12 (100%)
		S	12 (100%)
	CD8 (CD69+CD137+) N=11	M	9 (81.8%)
		N	11 (100%)
		S	8 (72.7%)
6-9 months from infection	CD4 (CD25+OX40+) N=8	M	7 (87.5%)
		N	8 (100%)
		S	8 (100%)
	CD8 (CD69+CD137+) N=5	M	4 (80%)
		N	4 (80%)
