# Supplementary material for: Lasting Changes to Circulating Leukocytes in People with Mild SARS-CoV-2 Infections
Source: Viruses. 2021 Nov 8;13(11):2239. doi: 10.3390/v13112239 (PMC8622816; doi:10.3390/v13112239)
Supplement: Supplementary file 1 [file viruses-13-02239-s001.zip › viruses-1441062-supplementary Table S4.pdf]

Supplementary Table S4. All immune parameter data analysis*

Welch's One-Way ANOVA with Games-Howell post-hoc: Significant factors are bold										
					post-hoc tests					
					“Other Illness" vs. "1-3 mo Post-COVID"		"Other Illness" vs. "6-9 mo Post-COVID"		"1-3 mo Post-COVID" vs. "6-9 mo Post-COVID"	
	F	df1	df2	p	Mean Diff.	P	Mean Diff.	p	Mean Diff.	p
**Percentage of CD45+ Events**										
Neutrophils	1.4707	2	19.6	0.254						
PBMCs	1.4511	2	19.6	0.258						
Total Monocytes	1.4251	2	16.8	0.268						
Classical Monocytes	1.7556	2	16.9	0.203						
Intermediate monocytes	0.3266	2	13.2	0.727						
Non-classical monocytes	0.8169	2	18.5	0.457						
**NK cells**	**3.6666**	**2**	**18.2**	**0.046**	**-3.19**	**0.043**	**-1.72**	**0.264**	**1.46**	**0.538**
B cells	0.0321	2	17.1	0.968						
CD3+ cells	0.8973	2	18	0.425						
CD4+ T cells	1.507	2	17.9	0.248						
CD8+ T cells	0.4834	2	15.8	0.626						
FoxP3+ T cells	2.163	2	17.1	0.145						
**Absolute Counts/mL Blood**										
CD45+ cells	2.3637	2	19.3	0.121						
Neutrophils	3.3724	2	18.7	0.056						
PBMCs	3.2755	2	17.8	0.061						
Total Monocytes	1.5412	2	19.2	0.239						
Classical Monocytes	1.3707	2	19.3	0.278						
Intermediate monocytes	0.0399	2	15.4	0.961						
Non-classical monocytes	2.255	2	18.5	0.1224						
NK cells	2.6014	2	15.4	0.106						
B cells	1.075	2	18.7	0.361						
CD3+ cells	0.2733	2	19.2	0.764						
CD4+ T cells	0.3703	2	17.4	0.696						
CD8+ T cells	0.1949	2	18.7	0.825						
FoxP3+ T cells	3.6161	2	11.8	0.059						
**Percentages of Monocytes**										
Classical monocytes	0.3733	2	17.2	0.694						
Intermediate monocytes	0.1175	2	14.2	0.890						
Non-classical monocytes	2.481	2	18.4	0.111						
**Percentages of CD3+ Events**										
CD4+ T cells	0.1421	2	17.4	0.869						
CD8+ T cells	0.0869	2	17.9	0.917						
**FoxP3 T cells**	**3.9274**	**2**	**17.1**	**0.039**	**-0.669**	**0.0489**	**0.0991**	**0.95**	**0.7677**	**0.061**
**Ratios**										
Myeloid:Lymphoid ratio	2.1683	2	19	0.142						
Classical: intermediate monocyte ratio	0.2377	2	12.6	0.792						
Intermediate: non-classical monocyte ratio	1.9887	2	15.3	0.171						
Classical: Non-Classical Monocyte ratio	0.9269	2	17.2	0.415						
CD4:CD8	0.348	2	14.2	0.712						
**Mean Fluorescence Intensity (MFI) Monocyte Population**										
**Total monocytes**										
**CX3CR1**	**5.7669**	**2**	**16.9**	**0.012**	**3473**	**0.022**	**773**	**0.829**	**-2700**	**0.053**
HLADR	2.9114	2	18.1	0.08						
**CCR2**	**4.7323**	**2**	**19.5**	**0.021**	**37964**	**0.338**	**-29177**	**0.449**	**-67141**	**0.014**
**CD11b**	**5.7993**	**2**	**14.1**	**0.015**	**-13929**	**0.012**	**-4786**	**0.464**	**9143**	**0.232**
**Classical Monocytes**										
**CX3CR1**	**6.2158**	**2**	**16.9**	**0.009**	**3146**	**0.023**	**346**	**0.956**	**-2800**	**0.032**
HLADR	3.5579	2	17.3	0.051						
CCR2	3.1883	2	19.3	0.064						
**CD11b**	**5.7515**	**2**	**14.1**	**0.015**	**-14307**	**0.012**	**-4744**	**0.482**	**9563**	**0.218**

**Intermediate Monocytes**										
CX3CR1	0.8799	2	17	0.433						
HLADR	0.7349	2	16	0.495						
**CCR2**	**7.1876**	**2**	**14**	**0.007**	**54305**	**0.24**	**-8934**	**0.916**	**-63239**	**0.016**
CD11b	3.1132	2	16	0.072						
**Non-classical monocytes**										
CX3CR1	0.9032	2	17.8	0.423						
HLADR	2.6647	2	17.4	0.098						
**CCR2**	**7.451**	**2**	**12.9**	**0.007**	**11039**	**0.03**	**-3513**	**0.829**	**-14552**	**0.045**
**CD11b**	**4.8731**	**2**	**15.4**	**0.023**	**-6787**	**0.023**	**-2875**	**0.319**	**3912**	**0.351**
Neutrophil CD11b	2.6824	2	17.4	0.097						
**Phenotype of CD4+ T cells as Percentage of CD4+ Events**										
Naïve	2.6536	2	18.7	0.097						
**Central Memory**	**5.1728**	**2**	**18.2**	**0.017**	**-13.6**	**0.009**	**-6.56**	**0.329**	**6.99**	**0.27**
Effector Memory	2.3065	2	19	0.127						
EMRA	0.4312	2	19.7	0.656						
Terminally Differentiated	2.8999	2	11.9	0.094						
**Phenotype of CD4+ T cells as Absolute Counts/mL Blood**										
Naïve	0.9228	2	18.6	0.415						
Central Memory	1.5868	2	15.9	0.235						
Effector Memory	0.0603	2	19.6	0.942						
EMRA	0.8185	2	17.4	0.457						
Terminally Differentiated	2.754	2	11.1	0.107						
**Phenotype of CD8+ T cells as Percentage of CD8+ Events**										
Naïve	0.7236	2	16.2	0.5						
Central Memory	0.5122	2	19.5	0.607						
Effector Memory	3.0705	2	17	0.073						
EMRA	0.6319	2	14.9	0.545						
**Terminally Differentiated**	**3.39**	**2**	**13.8**	**0.048**	**-2.949**	**0.038**	**-1.256**	**0.6**	**1.693**	**0.378**
**Phenotype of CD8+ T cells as Absolute Counts/mL Blood**										
Naïve	0.1872	2	19.1	0.831						
Central Memory	0.7365	2	19.3	0.492						
Effector Memory	0.5	2	17.9	0.615						
EMRA	1.1601	2	17.4	0.337						
Terminally Differentiated	2.822	2	13.8	0.076						
**Mean Fluorescence Intensity (MFI) CD4+ Population**										
**OX40**	**4.9848**	**2**	**16.15**	**0.021**	**-4175**	**0.018**	**46.6**	**0.999**	**4221.1**	**0.046**
**CCR6**	**6.2453**	**2**	**16.06**	**0.01**	**-1132**	**0.01**	**-518**	**0.163**	**613**	**0.244**
**CD69**	**5.2759**	**2**	**15.12**	**0.018**	**-11130**	**0.016**	**916**	**0.961**	**12046**	**0.033**
**CCR4**	**4.8032**	**2**	**16.36**	**0.023**	**-2590**	**0.016**	**-725**	**0.554**	**1865**	**0.13**
CXCR3	0.8101	2	17.87	0.461						
CD137	2.7273	2	11.21	0.108						
CD25	0.8736	2	17.97	0.434						
CD39	1.2827	2	15.8	0.305						
**Mean Fluorescence Intensity (MFI) CD8+ Population**										
OX40	1.582	2	14.65	0.223						
CCR6	1.0865	2	17.29	0.359						
CD69	2.6572	2	15.3	0.102						
CCR4	0.6502	2	15.36	0.536						
CXCR3	1.4059	2	14.21	0.277						
CD137	1.888	2	16.14	0.183						
CD25	1.0541	2	16.45	0.371						
CD39	0.7512	2	14.66	0.489						

*post hoc analysis included when significant.
