# Supplementary material for: Lasting Changes to Circulating Leukocytes in People with Mild SARS-CoV-2 Infections
Source: Viruses. 2021 Nov 8;13(11):2239. doi: 10.3390/v13112239 (PMC8622816; doi:10.3390/v13112239)
Supplement: Supplementary file 1 [file viruses-13-02239-s001.zip › viruses-1441062-supplementary Table S5.pdf]

Supplementary Table S5. Monocyte surface receptor expression as Mean Fluorescence Intensity (MFI)

			No Covid Infection	1-2 months post COVID infection	6-9 months post COVID infection	Statistical Significance
**Total Monocytes**	**CX3CR1**	Mean	14659	11187	13886	**0.012**
		SD	3328	2255	2411	
	**CCR2**	Mean	284856	246892	314032	**0.021**
		SD	65721	64926	36056	
	**CD11b**	Mean	8288	22217	13074	**0.015**
		SD	4180	14215	10348	
**Classical Monocytes**	**CX3CR1**	Mean	13459	10313	13113	**0.009**
		SD	3016	2102	2274	
	**CCR2**	Mean	344941	299049	351287	0.064
		SD	45175	62347	38683	
	**CD11b**	Mean	8342	22649	13086	**0.015**
		SD	4310	14619	10530	
**Intermediate Monocytes**	**CX3CR1**	Mean	32020	28127	31114	0.433
		SD	9414	6501	6844	
	**CCR2**	Mean	118366	64061	127299	**0.007**
		SD	63714	28494	51585	
	**CD11b**	Mean	14043	26926	18178	0.072
		SD	7902	16191	13349	
**Non-Classical Monocytes**	**CX3CR1**	Mean	45429	42083	45711	0.423
		SD	7431	7233	6917	
	**CCR2**	Mean	20651	9612	24164	**0.007**
		SD	11739	4882	13602	
	**CD11b**	Mean	7443	14230	10319	**0.023**
		SD	2487	8066	4865	
